# Sustaining complex interventions in long-term care: a qualitative study of direct care staff and managers

**DOI:** 10.1186/s13012-016-0454-y

**Published:** 2016-07-16

**Authors:** Cathleen Colón-Emeric, Mark Toles, Michael P. Cary, Melissa Batchelor-Murphy, Tracey Yap, Yuting Song, Rasheeda Hall, Amber Anderson, Andrew Burd, Ruth A. Anderson

**Affiliations:** 1 Duke University School of Medicine, Box 3003 DUMC, Durham, NC 27710 USA; 2 Durham VA Geriatric Research Education and Clinical Center, 508 Fulton St., Durham, NC 27705 USA; 3 University of North Carolina School of Nursing, Carrington Hall CB #7460, Chapel Hill, NC 27599 USA; 4 Duke University School of Nursing, 307 Trent Dr, Durham, NC 27710 USA

**Keywords:** Long-term care, Sustainability, Qualitative research, Nursing education research, Quality improvement, System intervention, Behavior change

## Abstract

**Background:**

Little is known about the sustainability of behavioral change interventions in long-term care (LTC). Following a cluster randomized trial of an intervention to improve staff communication (CONNECT), we conducted focus groups of direct care staff and managers to elicit their perceptions of factors that enhance or reduce sustainability in the LTC setting. The overall aim was to generate hypotheses about how to sustain complex interventions in LTC.

**Methods:**

In eight facilities, we conducted 15 focus groups with 83 staff who had participated in at least one intervention session. Where possible, separate groups were conducted with direct care staff and managers. An interview guide probed for staff perceptions of intervention salience and sustainability. Framework analysis of coded transcripts was used to distill insights about sustainability related to intervention features, organizational context, and external supports.

**Results:**

Staff described important factors for intervention sustainability that are particularly challenging in LTC. Because of the tremendous diversity in staff roles and education level, interventions should balance complexity and simplicity, use a variety of delivery methods and venues (e.g., group and individual sessions, role-play/storytelling), and be inclusive of many work positions. Intervention customizability and flexibility was particularly prized in this unpredictable and resource-strapped environment. Contextual features noted to be important include addressing the frequent lack of trust between direct care staff and managers and ensuring that direct care staff directly observe manager participation and support for the program. External supports suggested to be useful for sustainability include formalization of changes into facility routines, using “train the trainer” approaches and refresher sessions. High staff turnover is common in LTC, and providing materials for new staff orientation was reported to be important for sustainability.

**Conclusions:**

When designing or implementing complex behavior change interventions in LTC, consideration of these particularly salient intervention features, contextual factors, and external supports identified by staff may enhance sustainability.

**Trial registration:**

ClinicalTrial.gov, NCT00636675

## Background

As complex interventions are tested and adopted within long-term care (LTC), researchers and practitioners must carefully consider the issue of sustainability once the initial intervention or training phase is complete. Sustainability has been defined as the continued use of program components and activities for the continued achievement of desirable program and population outcomes [1]. Sometimes referred to as “routinization” or “institutionalization,” the concept of sustainability incorporates continued benefits, activities, and capacity of the organization to use the intervention effectively [2]. However, the sustainability of complex, evidence-based programs within organizations is challenging [3, 4], with self-reported continuation often less than 50 % [5]. Not surprisingly, a majority (65 %) of implementation science frameworks include sustainability as a key step [6].

While sustainability is a critical consideration for complex interventions, very little literature is available to guide those wishing to promote it. A 2012 systematic review found only 125 articles pertaining to intervention sustainability in healthcare, with none in the LTC setting [2]. We identified only one subsequent article pertaining to the sustainability of complex interventions in LTC; in this study following an 18-month intervention to improve the quality of palliative care in nursing homes in Scotland, a lower level of external support from a nurse specialist (less than half of the intervention level) resulted in maintenance of quality gains in most outcomes. However, outcomes declined substantially in facilities experiencing leadership turnover during the sustainability phase [7]. In the US LTC market, where resources to provide dedicated program staff are scarce and leadership turnover rates can exceed 100 % per year [2], additional data to guide sustainability planning is urgently needed.

We previously reported results from a randomized pilot study showing that an educational intervention, CONNECT, improved measures of staff communication and quality of care, with a trend to decreased facility fall rates [8]. As part of an ongoing randomized controlled trial of CONNECT in a larger sample of facilities, we conducted focus groups of direct care staff (nurse aides, dietary aides, social workers, activity staff, housekeepers, and nurses engaged in daily resident care) and managers (administrators, department managers, staff in education, or supervisory roles) to elicit their views on intervention sustainability. Although the effectiveness of this particular intervention is still under evaluation and consideration of sustainability therefore premature, these focus groups were an opportunity to identify barriers and facilitators to sustaining complex interventions more generally that may inform other LTC researchers and practitioners. Therefore, our goals for this analysis were (1) to obtain staff perceptions of intervention features that optimize or diminish sustainability of a complex intervention and (2) to identify processes, tools, and materials that promote continuation of behavior change interventions such as CONNECT in LTC facilities.

## Methods

The design of the parent study has been described previously [8, 9]. Briefly, the overall study tests a multicomponent staff education intervention based on complexity science (CONNECT) to promote new connections, information flow, and shared problem-solving about clinical issues among staff in LTC. Staff working in all capacities participated (e.g., dietary aides, nurse aides, housekeepers, nurses, rehabilitation staff, social workers, activity staff, department managers, and administrators). Facilities (*n* = 16) were randomized to receive either CONNECT for 3 months followed by the Falls Management Program [10] (Agency for Healthcare Quality Research’s quality improvement program) for 3 months or the Falls Management Program alone. The primary outcome is adjusted facility fall rate, with results expected in late 2016.

The CONNECT intervention included multiple components. During two group sessions, staff were introduced to the concept of local interaction strategies (LIS), which are ways of interacting with co-workers, and practiced using them to promote connection, information flow, and problem-solving. The intervention was delivered by trained Bachelors- and Masters-level research staff who did not necessarily have a clinical background. These group sessions used storytelling, role-plays, and interactive games to introduce LIS, learn the rationale for using them, and facilitate practice. Individual staff then met with researchers to create a personalized map showing specific co-workers with whom they wished to share more information about care of their set of residents; they used this map as a guide for completing a self-monitoring tool indicating their use of local interaction strategies over 6 weeks. Over time, staff received individual feedback and mentoring about their reported use of local interaction strategies. In two additional group sessions, department managers, without the administrator or Director of Nursing present, created current and ideal interaction maps for work groups as a whole across the facility and identified ways to improve group connections.

Staff in the eight facilities that were randomized to receive CONNECT participated in the present qualitative study focused on sustainability. Methods for this focus-group study are described in detail below.

### Qualitative design and participants

Focus groups (*n* = 15 groups) were conducted with 83 study participants in eight facilities following participation in CONNECT. Participants were purposefully selected based on their level of participation in the intervention and role in the facility (e.g., manager or direct care) to maximize diversity of perspective [11]; participants who had attended at least the first in-class session and were present in the facility on the day of the focus group were invited to attend. Focus groups were conducted within 1 month of the completion of the interventions. Group size was targeted at 8–12, and where possible, two separate focus groups were conducted with direct care workers and managers to optimize participant openness. Two study team members who were experienced in qualitative research and who had not been part of intervention delivery conducted the focus groups. Focus groups were convened in a private location (i.e., conference room) in the facility during regular working hours. All participants provided individual written informed consent. Ethics approval for this study was obtained from the Duke University Institutional Review Board, study number 18745.

Facilitators used an interview guide to elicit participants’ perceptions of whether and how CONNECT could be sustained in their facility. Interview guide questions and probes are listed in Table 1. Sessions were audio-recorded and professionally transcribed.

**Table 1:** Focus group questions, with analysis domains and intended charting category

Question	Codes	Domain
Grand tour question		
What was most important to you in the CONNECT program? What struck you about CONNECT?		
Probes		
How did the CONNECT program change the way you communicate about resident care?	• Change in communication	Salience of intervention components
Which parts of CONNECT, if any, had the biggest impact on the way you communicate about resident care? How? Examples.	• High impact • Resident care example	
Which parts of CONNECT, if any, were less helpful? Why? Examples.	• Low impact	
What would you change about how CONNECT was presented or delivered (for example, classroom sessions vs. self-study materials, vs. one-on-one discussions with CONNECT champions)?	• Suggested change	
Overall, what (if any) parts of the CONNECT program will you continue to do in your facility now that we have finished the program?	• Plan to continue	Sustainability
What can your facility do to continue using the CONNECT program? How will they refresh, update, and orient staff to CONNECT?	• Facility tailoring	
Now that we have talked about what pieces of CONNECT you want to keep and how you want to share them, what kinds of materials or tools do you need to share that information effectively?	• Tools needed	

### Framework analysis

Interview transcripts were analyzed using ATLAS.ti [12]. Framework analysis, a systematic process for qualitative data analysis, was employed to identify core concepts emerging from the focus groups. This analysis approach is well suited for cross-sectional data [13, 14], is particularly useful in healthcare research as it allows for a priori concepts (e.g., salience, sustainability) [15] to be combined with inductive analyses, and creates an explicit audit trail in the data reductions within and between individual facilities and between analytic stages [15, 16].

The analysis proceeded in five stages: familiarization, identifying thematic framework, coding, charting, and mapping and interpretation. In the familiarization stage, transcripts of each focus group were created with names and site numbers redacted to blind coders to the identity of the participant and site. All team members read all transcripts. In the second stage, a thematic framework was identified. We had two a priori domains directly related to our research questions: *salience*, or the aspects of the intervention staff perceived as more or less impactful, and *sustainability*, or staff descriptions of whether and how the intervention could be continued after the end of the study. At a team meeting, preliminary codes were defined based on interview guide questions and probes (listed in Table 1) and initial reading of the transcripts. In the third stage, each of the transcripts was coded (i.e., indexed) by at least two team members using the a priori codes. In addition, open coding was employed for the first four transcripts and emerging themes were added in an iterative manner. In the fourth charting phase, we rearranged the data so that all quotes indexed with the same code were grouped together for each facility. The data were distilled during this step; for each coded quotation, a team member developed a brief summary statement, which linked the quote to the research questions. The charting process was repeated to synthesize the individual quote summaries into facility-level summaries (e.g., a summary of facility D’s quotes with the code “high impact”) and to create summaries for each code by manager and direct care focus group types (e.g., a summary of all manager group’s quotes with the code “suggested change”). At least two team members reviewed and validated the charting at each step. Disagreements were discussed during team meetings. Confirming and disconfirming evidence was sought from the primary data. In the final mapping and interpretation phase, we used the charts to describe key insights and compare them across facilities and group roles. For the presentation of findings, we took this highly synthesized set of findings from the mapping phase (step 5) and organized them using prior conceptual work by Scherier [1]. Scherier’s work suggests that sustainment of interventions that require coordination among multiple staff members is strongly influenced by three factors: characteristics of the intervention itself, factors within the organizational context, and external supports.

## Results

Characteristics of the study facilities and focus group participants are listed in Table 2. In the sections that follow and in Table 3, we describe key insights from LTC staff descriptions of factors affecting sustainability in the categories of intervention features, organizational context, and external supports.

**Table 2:** Characteristics of study facilities and focus group participants

Nursing home	*N* = 8	
Bed size (mean)	130	
For profit (%)	100	
Urban location (%)	50	
Study participants	Managers *N* = 34	Direct care staff *N* = 49
Age (%)		
18–35 years	22	39
36–55 years	62	51
56 years and older	16	10
Female (%)	84	93
Race (%)		
Caucasian	72	25
Black	25	63
Others	3	12
College or Associate degree (%)	91	32
Role (%)		
Administrator	5	0
Department manager	59	0
Social work	12	0
Nurse aide	0	43
Direct care nurse	0	25
Dietary	12	0
Housekeeping	0	16
Rehabilitation	12	2
Others	0	14

**Table 3:** Key LTC staff insights about sustainability and potential approaches suggested by focus group participants, CONNECT study experience, or implementation science literature

Key participant insights	Potential approaches
Intervention features	
Content	
• Must be perceived to be beneficial and promote organizational aims • Must balance complexity and simplicity	• Include regular outcome measurement with participant feedback • Reinforce impact on resident outcomes • Pilot content with full range of target staff
Delivery	
• Group sessions allow mutual instruction, increase confidence, give “permission” to bring up problems, and strengthen relationships • Individual sessions allow assessment of understanding and customization • Trainers should balance clarity and excess repetition • Reinforcement and practice of new skills is needed	• Consider combination of group and individual sessions for interventions requiring staff coordination • Use role-play, storytelling, and other means to promote interaction • Use mentored practice sessions with feedback
Customizability/flexibility	
• Sessions should accommodate clinical demands, include all shifts, and be customized to fit each facility’s schedule	• Build flexibility into intervention testing, e.g., allow staff to choose when/where instruction occurs, number of sessions (multiple short vs. single long), number of participants per session • Test number of “booster sessions” needed to sustain desired level of change
Materials	
• Intervention materials should consider diversity of staff; make learning objectives pertinent regardless of role, experience, education level, language • Materials should be visually appealing and in different formats	• Use range of authentic case scenarios of interdisciplinary interest when possible • Use graphics, stories that are understandable to diverse target audience • Consider range of print, online, video
Contextual features	
Leadership	
• Direct care staff want to observe active leadership support and engagement in the program • Lack of trust and gaps in communication frequently exists between direct care staff and managers	• Avoid always separating managers and staff for training • Consider whether manager/direct care staff communication issues can be addressed as part of the intervention (e.g., promote discussion, include team building approaches)
Incentives	
• “Accountability” to change behavior is expected by staff • Desired behavior should be an expected part of the culture	• Avoid mandatory training sessions • Avoid rule-based, “shame and blame” approach, but instead articulate shared goals, vision to be accomplished by sustaining program, particularly impact on residents
External supports	
Processes and procedures	
• Formalizing changes through changing work routines promotes continuation • New staff orientation is a key target for continuing training • “Refresher” sessions are needed • Use approaches such as “train the trainer,” facility champions to promote continuation	• Make explicit changes to meeting schedules, documentation templates, work rounds, etc. • Facilitate changes to orientation schedule • Incorporate champion training midway into intervention
Tools	
• Visual aids and reminders scattered throughout work environment are helpful • Address training for those unable to attend in-person sessions • Creative uses of information technology are now feasible in LTC	• Develop posters, bulletin boards, bookmarks, calendars, pens, etc. • Develop orientation package • Consider use of DVDs, video clips, web-based training sessions followed by individual/small group discussions • Reminders within electronic medical record, online training resources

### Intervention content

LTC staff reported that sustainable intervention content must be *perceived to be effective* and to *promote organizational aims*. Specifically, both direct care staff and managers across facilities reported being highly motivated to continue the behavior change when they perceived it would improve care for their residents.

RN: For me there are some people that are unapproachable…but this [program] just kind of helped me to approach them for the benefit of the resident…Even if I didn’t want to talk to that person I had to try to make it work so it would just be about the residents. So I liked how [the intervention] focused back on the residents.

The *complexity* of the intervention was also reported to influence how likely it was to be adopted and therefore sustained. The staff comments also highlighted the challenging balance for interventions targeting staff from a wide range of disciplines; a professional staff member in one facility reported that the intervention was too simplistic, while two nurse aides in another facility reported being confused about parts of the intervention.

SPEECH THERAPY: It was pretty basic, just kind of a review… I don’t think I got any new information or felt like I was inspired to do anything different.

NURSE AIDE: I’ve been very confused in the [class session] because I wasn’t told what the map was for…So I just randomly just gave some names because that’s what my instructions were to me.

*Intervention delivery* was reported to be important for its uptake and sustainability, with advantages and disadvantages perceived with different formats. Staff reported that *group sessions* allowed participants to learn from each other, build confidence about their importance to resident care, give “permission” to speak out about problems in the facility, and strengthen personal relationships. *Role-playing* was cited as a particularly effective delivery method within group sessions.

STAFF DEVELOPMENT COORDINATOR (SDC): You need to do the group thing… because everybody is going to have different opinions and you want people to listen and take something from that other person.

NURSE AIDE: I feel like I’ve gotten the confidence to build more relationship with whatever nurse I’m working with… I have more confidence to go to them, just by knowing that’s okay.

SOCIAL WORKER: And since [the group session]… I spoke out about some things that I didn’t think we’re doing quite well.

On the other hand, *individual sessions* allowed material to be customized and clarified and reinforced key concepts. Staff valued opportunities to *practice and monitor new skills*.

SPEECH THERAPY: I really liked keeping track of who I talk to because I would start to want to talk to them more and say “well I haven’t [used the intervention] with them yet.”

Staff across facilities emphasized that in the busy long-term care environment, the intervention needed to be *customizable and flexible*. They appreciated that sessions could be delivered in multiple locations and formats (e.g., multiple shorter vs. fewer longer sessions, classroom vs. nursing station) and worked around clinical demands.

SDC: You guys…did have an open schedule and that was good. And you did allow some variations with the timing with the people which was good. You just have to play it by ear. (Laughing) There is no set time, there is no set schedule because everyone is extremely busy and things come up.

In regard to educational *materials*, the participants encouraged development of visually appealing materials in different formats (e.g., print, video, online) to accommodate different learning styles. Non-nursing staff urged creating materials relevant to other types of roles in the facility (e.g., scenarios inclusive of dietary assistants and environmental service workers).

DIETARY DIRECTOR: Some of my staff, they did not sign up because most of the stuff was centered around nursing.

### Organizational context

In the highly *regulated* LTC industry, staff warned that sustainability cannot occur when the program becomes just another mandatory training program or “chore.”

SDC: We’ve got a program now that all the staff has to do and I have to… make sure they’ve got it done…in a timely manner each month. So it’s just another chore. It’s not as much a learning situation as it’s a chore; “Oh I’ve got to get that done because [otherwise] I can’t work.”

However, suggestions for how to create appropriate *incentives* for sustainability were sparse. Several direct care staff members talked about creating “accountability” for participation by making participation mandatory.

NURSE AIDE: See, it was all voluntary. It wasn’t mandatory and so everybody was just “Okay, I’m not going since it’s just voluntary.”… If this was mandatory, maybe your point would get across and more people would participate.

A single social worker expressed the idea of *changing organizational culture* to sustain change but without specific suggestions on how to achieve it.

SOCIAL WORKER: Yeah and how we can hold ourselves accountable except for when we just came to the meeting? But when we were out [of the classroom] as a staff were we supportive of [the program] and was that part of our culture, do we take it into our culture?

In four facilities, staff agreed that an important part of sustaining the intervention was ensuring *leadership support*. In these facilities, direct care staff described a deep divide between managers and direct care workers that impacted whether or not the intervention was perceived as a priority.

SOCIAL WORKER: The leadership [needs to buy in]. NURSE AIDE: Right, ‘cause that’s how it is. The leader [has] got to lead for us to follow. They [are] just here for the money. We care more than them. SOCIAL WORKER: It’s normal to have attitudes [about your leaders] about where you’re working… A lot of time you feel like they didn’t really buy into it. So it really wasn’t a priority.

The CONNECT study provided a separate class for administrators and managers at the beginning of the intervention period, in an attempt to secure their early support and to prevent their presence from inhibiting direct care staff participation in classroom sessions. However, the fact that managers were not present with staff in the sessions was perceived as a barrier to uptake and sustainment because direct care staff concerns could not be aired and it appeared to them that managers lacked interest in the program.

NURSE AIDE 1: There were some nurses [in the session] but I think it would be helpful if we had like the Assistant Director of Nursing, Director of Nursing and possibly the Administrator. (Laughing in the room) NURSE AIDE 2: When we were doing [another class] we seen them in here but we didn’t see them when you guys were here though… NURSE AIDE 1: And there’s no possible way that ya’ll can get [the managers] together? Because that way they’ll be able to hear our concerns right in front of them.

Clearly, sustainability requires not only general leadership support for the program but also direct care staff seeing their active participation and prioritization. Interestingly, no discussion about the importance of leadership support occurred in the managers’ focus groups.

### External supports

In response to questions about what type of support would help them to sustain the intervention in their facility, staff identified a number of *processes and procedures* that could be adopted. At least one facility reported changing their *meeting and reporting structure* to include more direct care involvement. Staff in most facilities considered adding CONNECT training to be a standard part of *new employee orientation* to address the issue of frequent staff turnover. Several facilities suggested adopting a “train the trainer” approach, where *facility champions* are identified to encourage continuation and conduct periodic *refresher sessions*.

RECEPTIONIST: I think this would be something good for orientation for them to go over… and a refresher because we do have in-services every month.

DIRECTOR OF NURSING: We need… a CONNECT champion that continues to keep people updated.

*Tools* that could support sustainment were suggested including *posters or other visual reminders* such as pens and calendars of bulletin boards. *DVDs or video clips* for staff who cannot attend a class were suggested. *Orientation materials* for new staff were suggested in all eight facilities given high staff turnover.

REHABILITATION DIRECTOR: Or a two minute [video clip] of a nurse coming in and dealing with a couple other staff members and somebody gets upset about something. Everybody goes off and [views it]. But then you [get together and ask] how could this scenario have changed?

Finally, creative use of *information technology* embedded in electronic health records was suggested as a means to keep the program fresh for staff.

NURSE AIDE: That’s what we have to do all of our charting [on the computers]. So if something like that popped up, a little in-service on there, we could…read it, ‘cause we have to chart. We’re probably there two or three times a day, at least.

## Discussion

Work in healthcare settings other than LTC suggests that the sustainability of complex interventions relates to the innovation itself (fit, adaptability, effectiveness), the context (regulation, culture, structure), processes (e.g., alignment of the intervention and the setting), and the capacity to sustain (e.g., funding, resources, workforce characteristics, and stability) [2]. Within a randomized trial of a behavior change intervention, we used rigorous qualitative methods to elicit staff perceptions of sustainability of behavior change specific to LTC. Our findings confirm that each of these categories is important and provide specific illustrations or suggestions for operationalizing them in LTC. Some suggestions from our participants have been widely used in other settings, for example, using external supports such as clinical champions and orientation materials [17, 18]. However, other insights that relate to specific issues in LTC and require special consideration are discussed below.

Leadership support relating to sustainability requires particular attention in LTC, where historically median job tenure for administrators has been less than 1 year [2]. Ongoing stakeholder buy-in, supervision, and outcome monitoring have been identified as critical components for sustainability by others [5, 19–22]. Indeed, in a study of a national mental health program, the only facility-level factor associated (negatively) with sustainability was leadership turnover [19]. We propose potential strategies to obtain ongoing leadership support in the face of frequent turnover such as leveraging nursing home corporation-level policies (e.g., sustainability as a performance incentive for administrators), ongoing promotion through professional societies such as National Association of Directors of Nursing Administration—LTC, or identifying local champions with long tenure who can influence new leadership in the facility. These strategies need to be tested in future research.

Beyond turnover, however, participants in a majority of our study facilities identified pervasive mistrust and lack of communication between management and direct care staff, which has been previously observed in other LTC facilities [23, 24] and which was reported by our participants to impact their uptake and sustain implementation of CONNECT. While the parent randomized trial is ongoing and measures of the impact of CONNECT are not yet available, we observed that intervention participation rates were substantially lower and observed minimal changes in staff communication measures in facilities where staff reported this type of distrust between direct care staff and management. Therefore, it appears to be critical for both uptake and sustainment that leaders demonstrate their support of a program with visible, active participation; our well-intentioned separation of managers and staff in class sessions had the unintended consequence of aggravating the existing communication divide.

Incentives for sustained behavior change in LTC also need to be carefully considered. Several participants talked about the need for “accountability” to continue behavior change, referring to common LTC practices such as mandatory training, inspections, and penalties for failing to comply with workplace rules. While rule-based management approaches and “shame and blame” work environments are commonly used to develop accountability in LTC, both prior literature and some of our participants suggested that these are likely to be ineffective for sustaining behavior change [24]. Rather, the findings suggest that leaders might encourage institutionalization of the change into the work culture by articulating how it positively impacts shared goals and values. For example, our participants reported being particularly motivated to maintain practices that they believed benefited their residents.

Intervention-level factors that our participants identified as critical for sustainability in LTC included customization and flexibility. Implementation science has long recognized the importance of customization and the tension between continuing programs as originally designed versus the need to adapt them to make program components operational in new environments [2]. Participants in a majority of facilities valued the ability to tailor intervention delivery to accommodate various roles, shifts, and the frequent unforeseen circumstances that arise. In contrast to other healthcare settings with more predictable clinical demands (e.g., outpatient clinics) or higher staff to patient ratios (acute care), it is very challenging to have staff attend regular training sessions during working hours in LTC, and flexibility in how and when education occurs is especially critical. Behavior change interventions sometimes build flexibility into the design, but they rarely test what dose and frequency of “booster” interventions are necessary to sustain the desired level of change. Our study supports prior calls for investigators to clearly define sustainability in context, define outcomes or desired benefits, identify an appropriate measurement time frame, and study fidelity and adaptation [2]. Some investigators have argued that the complexity and heterogeneity of healthcare systems requires a non-linear approach to sustainability that integrates the themes of adaptive, contextually sensitive continuous quality improvement (CQI) and a learning healthcare system with the challenge of intervention sustainment [25]. The “Dynamic Sustainability Framework” argues that interventions must be adapted to fit within individual practice settings and its broader ecological system; since settings and systems change over time, so too must the intervention continuously evolve [20]. The implication is that “intervention optimization” must continue throughout the sustainability phase. This framework may be particularly salient for sustainability in the LTC setting.

Attention to diversity is another intervention-level factor identified by staff that is particularly challenging within LTC. Whereas care in hospital and outpatient settings is delivered primarily by licensed clinical staff with higher educational levels, in LTC, most direct care is delivered by unlicensed staff with high school or equivalent degrees. Behavior change interventions in LTC must therefore span a wider range of clinical expertise and educational levels. Diversity in long-term care also encompasses role/profession, literacy levels, race/ethnicity, and native languages. Intervention developers must use materials that are pertinent and accessible to this diverse target audience and determine a frequency of delivery that optimizes understanding while minimizing excessive repetition. For example, role-play activities in CONNECT were universally acceptable regardless of staff roles; an improvement would be to integrate stories that include a variety of staff roles into the role-play. This approach would better support inclusiveness of non-nursing staff in the learning sessions and could be used to address some of the communication gaps that also impact sustainability.

Our study also confirms findings of sustainability studies in other settings. In a multisite chronic care management intervention study in Sweden, intervention sites that showed the greatest improvement in the first year of the program also demonstrated the highest levels of sustainability [26]. In a study evaluating a teamwork-promoting intervention in emergency departments, groups that did not receive positive feedback from their behaviors did not sustain behavior changes [27]. These studies and our participant comments suggest that individuals are most likely to maintain programs when positive results are clear to them. Regular coaching and program evaluation with participant feedback has been reported to be effective in sustaining quality improvement interventions in home care [28] and was reported to be an effective way to provide feedback on effectiveness by some of our focus group participants. However, some participants wished that managers and not just research staff would acknowledge them for changing their behavior and believed this would have a broad-scale impact on uptake and sustainability.

These findings will be used by our research team in several ways. The main study results on CONNECT are expected in 2016, and if effective, the intervention will be streamlined to include the most salient elements identified by staff during the focus groups. Suggested tools (videos, training manuals) will be developed to facilitate widespread adoption, and intervention sustainability will be measured in a real-world pragmatic study. CONNECT is currently being adapted for use in other healthcare settings which require interprofessional team care within the Department of Veterans Affairs.

This qualitative study limited us to reporting perceptions of LTC staff about sustainability, rather than providing direct evidence of the effectiveness of sustainability approaches; it was a hypothesis-generating study identifying strategies that might be tested in future studies. It is important to note that the effectiveness of the complex intervention in the ongoing parent study, CONNECT, has not yet been fully established; staff perceptions of how helpful CONNECT was in their facility may have impacted their responses in the focus groups. We were limited to eight participating facilities in one region of the USA, which impacted the generalizability of our findings. Nevertheless, we believe that the results provide important insights that interventionists, practitioners, and administrators should consider when designing or deploying complex interventions in LTC. Practical tools to assist in designing sustainable interventions have been developed, such as the United Kingdom National Health System Institute for Innovation and Improvement Sustainability Model Tool to self-assess intervention-level, context-level, and external support factors [29]. Our study provides a rich context within which to interpret and extend these recommendations for LTC research and quality improvement. Additional research is needed to explicitly test sustainability approaches in LTC.

## Conclusions

LTC is a unique healthcare setting with particular challenges for intervention sustainability including diversity in staff role and educational level, frequent communication gaps or mistrust between direct care and managerial staff, and high leadership turnover. These factors must be considered when designing and implementing behavioral change interventions in LTC; the suggested approaches from staff described in this study may be helpful but require testing in future studies.

## Abbreviations

LTC, long-term care; SDC, staff development coordinator; UK, United Kingdom; USA, United States of America
